# Daily Orange Consumption Reduces Hepatic Steatosis Prevalence in Patients with Metabolic Dysfunction-Associated Steatotic Liver Disease: Exploratory Outcomes of a Randomized Clinical Trial

**DOI:** 10.3390/nu16183191

**Published:** 2024-09-20

**Authors:** Maria Notarnicola, Valeria Tutino, Valentina De Nunzio, Anna Maria Cisternino, Miriam Cofano, Rossella Donghia, Vito Giannuzzi, Marianna Zappimbulso, Rosa Anna Milella, Gianluigi Giannelli, Luigi Fontana

**Affiliations:** 1 Laboratory of Nutritional Biochemistry, National Institute of Gastroenterology—IRCCS “Saverio de Bellis”, Castellana Grotte, 70013 Bari, Italy; valeria.tutino@irccsdebellis.it (V.T.); valentina.denunzio@irccsdebellis.it (V.D.N.); miriam.cofano@irccsdebellis.it (M.C.); 2 Ambulatory of Clinical Nutrition, National Institute of Gastroenterology—IRCCS “Saverio de Bellis”, Castellana Grotte, 70013 Bari, Italy; annamaria.cisternino@irccsdebellis.it; 3 Data Science Unit, National Institute of Gastroenterology—IRCCS “Saverio de Bellis”, Castellana Grotte, 70013 Bari, Italy; rossella.donghia@irccsdebellis.it; 4 Unit of Gastroenterology, National Institute of Gastroenterology—IRCCS “Saverio de Bellis”, Castellana Grotte, 70013 Bari, Italy; vito.giannuzzi@irccsdebellis.it (V.G.); marianna.zappimbulso@irccsdebellis.it (M.Z.); 5 Research Centre for Viticulture and Enology, 70010 Turi, Italy; rosaanna.milella@crea.gov.it; 6 Scientific Direction National Institute of Gastroenterology—IRCCS “Saverio de Bellis”, Castellana Grotte, 70013 Bari, Italy; gianluigi.giannelli@irccsdebellis.it; 7 Charles Perkins Centre, Faculty of Medicine and Health, The University of Sydney, Sydney, NSW 2006, Australia; luigi.fontana@sydney.edu.au; 8 Department of Endocrinology, Royal Prince Alfred Hospital, Sydney, NSW 2050, Australia

**Keywords:** MASLD, phytochemical food, CAP, steatosis, oranges

## Abstract

**Background**: Consumption of flavonoid-rich orange juice has been shown to reduce adiposity and liver steatosis in murine models of diet-induced obesity. However, little is known about the effects of whole orange intake, independent of body weight changes, on liver function and steatosis in individuals with metabolic dysfunction-associated steatotic liver disease (MASLD). The goal is to understand the direct impact of orange consumption on metabolic health. **Methods**: Sixty-two men and women aged 30–65 with MASLD (Controlled Attenuation Parameter, (CAP) > 275 dB/m) were randomly assigned to consume either 400 g of whole oranges or non-citrus fruits daily for 4 weeks. Baseline evaluations included medical assessments, blood tests, and body composition. Liver health was assessed using transient elastography (FibroScan^®^) for steatosis and fibrosis, conducted by blinded personnel. This clinical trial was registered at ClinicalTrials.gov (NCT05558592). **Results**: After 4 weeks of orange supplementation, liver steatosis decreased in the treatment group, with 70.9% showing steatosis compared to 100% in controls (*p* < 0.004), indicating a 30% reduction in liver disease prevalence. There were no significant changes in fibrosis or plasma liver enzymes, though plasma gamma glutaril transferase (GGT) levels decreased significantly. Body weight, waist circumference, body composition, lipid profile, fasting glucose, insulin, and C-reactive protein levels remained unchanged. Dietary analysis revealed no change in caloric intake, but vitamins C, A, thiamine, and riboflavin increased in the orange group. **Conclusions**: Our findings suggest that phytochemical-rich foods, especially whole fruits like oranges, may enhance liver function as an adjunct treatment for MASLD. The notable reduction in liver steatosis prevalence occurred independently of body weight changes. Further studies are needed to investigate the long-term effects of orange supplementation on steatosis and fibrosis progression and to identify the specific bioactive compounds and mechanisms involved.

## 1. Introduction

Diets enriched with flavonoids have been closely associated with the prevention and treatment of various metabolic diseases, including obesity, dyslipidemia, insulin resistance, hepatic steatosis, type 2 diabetes, cancer, and atherosclerosis. 

Flavonoids exhibit potential health benefits due to their antioxidant, anti-inflammatory, and hypolipidemic properties, making them promising nutraceutical agents for handling various pathological conditions [1,2,3].

Flavonoids are phytochemicals found abundantly in vegetables and fruits, particularly in citrus fruits, especially in the albedo and membranes separating orange segments [1]. Key flavonoids include narirutin, naringenin, and tangerine, with hesperidin being the major component. Known for their antioxidant, anti-inflammatory, and hypolipidemic properties, flavonoids have been hypothesized to offer significant metabolic health benefits [2,4].

Moreover, in vitro, flavonoids extracted from oranges of the “Tacle” variety have demonstrated an inhibitory action on cholesterol synthesis and biomarkers levels involved in inflammation [5]. Under normal conditions, adipose tissue stores lipids in the form of triglycerides, whereas during obesity, hyperlipidemia causes excessive infiltration by macrophages in the adipose tissue and liver, resulting in the production of proinflammatory cytokines, such as tumor necrosis factor (TNF-α), interleukin 6 (IL-6), and inducible nitric oxide synthase (iNOS) [6,7], associated with systemic inflammation and atherogenesis [8,9].

For instance, protocatechuic acid administration for 10 weeks reduced lipogenic enzyme expression and hepatic lipid accumulation in high-fat-diet mice [10]. Preliminary clinical trial data suggest that 2 weeks of orange juice consumption positively affects lipid metabolism, particularly triglyceride-specific fatty acid chains and cholesterol esters in individuals with obesity and insulin resistance [11]. However, the interpretation of data from these preclinical and clinical studies is often confounded by lifestyle and weight changes among subjects randomized to consume orange juice.

Consumption of juice from anthocyanin-rich oranges for 12 weeks has demonstrated multiple beneficial effects in murine models of diet-induced obesity, including preventing weight gain, improving insulin sensitivity, reducing serum total cholesterol and triglycerides, lowering liver enzymes, and reversing liver steatosis [12,13]. These benefits are believed to be mediated through the induction of peroxisome proliferator-activated receptor-α (PPAR-α) and its target gene acyl-CoA oxidase, a key enzyme involved in lipid oxidation. However, the specific effects of orange fruit intake, independent of weight loss, on individuals with metabolic dysfunction-associated fatty liver disease (MASLD) remain largely unknown, particularly concerning liver function, steatosis, and fibrosis as assessed by vibration-controlled elastography (VCTE) using FibroScan^®^.

Recent evidence suggests that hesperidin may potentially improve non-alcoholic fatty liver disease (NAFLD) by exerting hypoglycemic effects, promoting fatty acid β-oxidation through activating silent information regulator 1 (SIRT1)/ peroxisome proliferator-activated receptor gamma coactivator 1α (PGC1α), and, finally, modifying lipid profiles [14].

NAFLD, now known as metabolic dysfunction-associated fatty liver disease (MAFLD), represents the most common cause of chronic liver disease worldwide, with a 20–30% prevalence in Western countries [15].

MAFLD, unlike the term NAFLD, emphasizes metabolic risk and focuses on alterations in glucose (insulin resistance) and lipid metabolism (lipotoxicity, oxidative stress, etc.) as well as the significant role of inflammatory processes in hepatocytes [16,17].

In this scenario, the main aim of this randomized clinical trial was to investigate the effects of 4-week consumption of “Navelina” whole oranges, independent of weight changes, on metabolic and liver function in 62 middle-aged overweight men and women diagnosed with MAFLD.

To isolate the independent effects of orange supplementation on metabolic and liver function, each subject was contacted by one of the investigators at least once every week by phone to review their medical condition and reinforce the importance of maintaining their usual food intake and physical activity, ensuring stable body weight. We hypothesized that ingesting the entire fruit, including its flavonoid-rich albedo, would offer favorable effects on liver function and steatosis, independent of weight modifications. This focus is crucial as it aims to understand the direct impact of orange fruit consumption on metabolic health, removing the confounding variable of weight change.

## 2. Materials and Methods

### 2.1. Orange

The “Navelina” variety oranges used in this study were biological oranges purchased by a BioFarm from the Cosenza (Calabria Region, Italy).

The physicochemical properties, polyphenol content, and total antioxidant activity of oranges were analyzed by the Council for Agricultural Research and Economics (CREAVE), Turi, BA, Italy. Polyphenolic profiles were determined by high-performance liquid chromatography (HPLC) 1100 (Agilent Technologies, Palo Alto, CA, USA) equipped with a diode array detector (DAD) analysis (Table 1).

### 2.2. Participants

Seventy men and women aged 30–65 years diagnosed with MAFLD were recruited from the outpatient nutrition clinic of the National Institute of Gastroenterology “S. de Bellis” between February 2023 and November 2023. Inclusion criteria required participants to exhibit liver steatosis (CAP score > 275 dB/m) along with either overweight status (Body Mass Index, BMI > 25), type 2 diabetes, and/or metabolic syndrome. All of the subjects had had a stable weight (with fluctuations of no more than 2 percent of the body weight) for at least two months and had been sedentary (exercising for less than one hour per week) for at least six months before entering the study. Approval for this study was obtained from the Human Studies Committee of the IRCCS Oncological Hospital—Giovanni Paolo II, Bari, Italy (Approval Number #184 del 13 May 2022).

**Figure 1:**
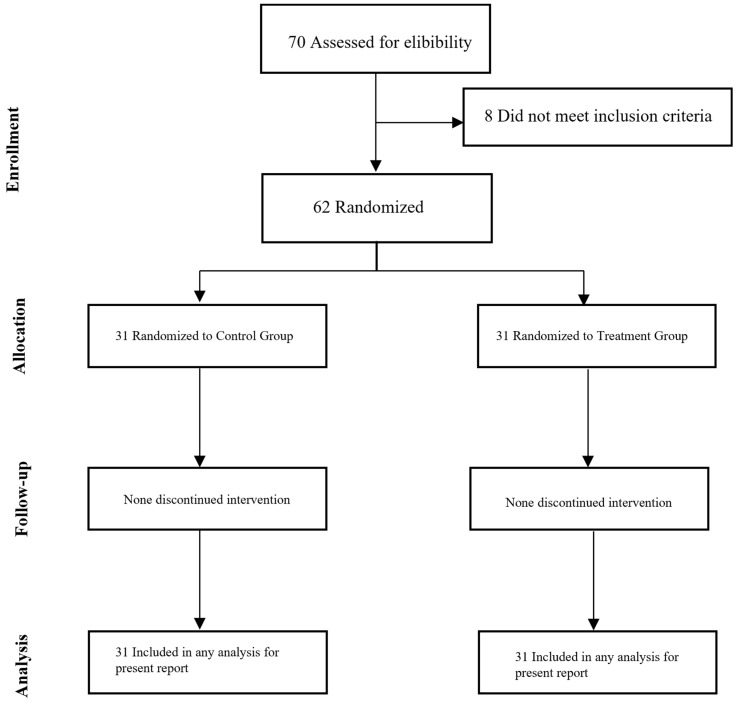
CONSORT flow diagram.

All participants provided informed consent following the principles outlined in the Declaration of Helsinki. The CONSORT diagram illustrating participant enrollment is depicted in Figure 1. This clinical trial was registered at ClinicalTrials.gov (NCT05558592). 

### 2.3. Study Design

At baseline, all participants underwent a comprehensive medical and nutritional assessment, including routine blood tests. Potential subjects were excluded if they had a history of gastroesophageal disease, chronic inflammatory disease, a recent history or evidence of malignancy, anticoagulant therapy use, or adherence to a special diet. Eligible participants were randomly assigned to one of two groups: consuming 400 g of oranges (net of waste) daily for 4 weeks or consuming 400 g of non-citrus fruits daily. The “Navelina” variety oranges used in this study were sourced organically from a BioFarm in the Calabria Region, Italy. The physicochemical properties, polyphenol content, and total antioxidant activity of the oranges were analyzed by the Research Center for Viticulture and Enology of Council for Agricultural Research and Economics (CREAVE) in Turi (BA), Italy. Analysis of polyphenolic profiles was conducted using high-performance liquid chromatography (HPLC) 1100 (Agilent Technologies, Palo Alto, CA, USA) equipped with a diode array detector (DAD) as shown in Table 1.

All study participants were provided with dietary guidelines, which included recommendations to restrict the intake of alcohol, caffeine, and foods rich in vitamin C (Supplementary Table S1). A total of 62 research volunteers (17 females and 45 males) began the intervention. Each subject was contacted by an investigator at least once a week via phone. During these calls, the investigator reviewed the participant’s medical condition, reinforced the importance of adhering to their usual diet and physical activity levels, and emphasized the necessity of maintaining a stable body weight throughout the study. This regular communication was designed to ensure that any observed effects could be attributed specifically to orange consumption rather than changes in weight or other lifestyle factors. All study personnel performing assessments were masked to treatment assignment. Participants were advised to maintain their usual dietary and physical activity habits throughout the study.

### 2.4. Anthropometrics and Dietary Assessment

Body weight was measured in duplicate in the morning following a 12 h fast with the subject wearing a hospital gown and no shoes. Height and waist circumference were measured twice using a wall-mounted stadiometer and tape measure. BMI was calculated as weight divided by the square of height (kg/m^2^). Changes in body weight and body fat mass were assessed by bioelectrical impedance analysis—BIA (BIA 101, Akern SRL, Pontassieve, Italy). The phase angle (PhA) was calculated using the arctangent of the extracellular water (ECW) ratio, directly derived from Rz and Xc values with Bodygram PLUS Software v. 1.0 (Akern SRL, Pontassieve, Italy) utilizing medically validated algorithms. Seven-day food diaries were used to estimate self-reported intake. Participants received detailed instructions on how to weigh, measure, and record all food and beverages consumed during the collection. Research dietitians reviewed the diaries with participants and then analyzed them using MetaDIETA Professional 4.0.1 (Meteda, Rome, Italy).

### 2.5. Blood Analyses

Venous blood was sampled for metabolic and hormone concentrations after an overnight fast. Samples were collected in serum and edetic acid plasma tubes, immediately centrifuged to separate the plasma, aliquoted, and stored in a −80 °C freezer until use. All serum and plasma samples were analyzed by the Core Laboratory at the National Institute of Gastroenterology “S. de Bellis”; technicians performing assessments were masked to treatment assignment. 

### 2.6. Liver Fibroscan^®^

Non-invasive transient elastography (FibroScan^®^, Echo-Sens, Paris, France) was performed to evaluate hepatic steatosis and fibrosis after fasting for at least 4 h. All FibroScan^®^ measurements were taken by highly trained technicians who were masked to treatment assignment. Steatosis was assessed by CAP (dB/m), and liver stiffness was measured in kPa.

### 2.7. Statistical Analysis

All participants who provided both baseline and 4-week data were included in the analyses. Data are presented as mean and standard deviation (M ± SD) for continuous variables at each time point and for the change between baseline and 4 weeks, while data are presented as frequency and percentages (%) for categories. Baseline characteristics were compared between groups with the Chi-square test employed for categorical variables as needed, while the Wilcoxon rank was used for continuous variables. The Wilcoxon matched-pairs signed-rank or McNemar’s test was applied for continuous or categorical parameters to evaluate variations after and before 4 weeks of observation. To test the association between the independent groups, the Chi-square test was employed for categorical variables as needed, while the Mann–Whitney test was used for continuous variables. All statistical tests were two-tailed, and significance was accepted at *p* < 0.05. All analyses were performed using StataCorp. 2023 software, version 18 (College Station, TX, USA: StataCorp LLC), while RStudio (“Chocolate Cosmos” Release) was used for the plots.

## 3. Results

### 3.1. Study Participants

A total of 70 participants were screened for eligibility, with 62 being randomized and starting the intervention. All participants completed the 4-week intervention (Figure 1). 

Subjects randomized to the orange supplementation group (*n* = 31; age 51.8 ± 10.3 years) and the control group (*n* = 31; age 50.1 ± 9.8 years) had similar baseline characteristics, except for higher plasma concentrations of total cholesterol and GGT levels and lower HDL cholesterol in the treatment group (Table 2). 

The analysis of an adherence questionnaire demonstrated compliance with the recommended diet. The macronutrients contained in the diet used and daily calorie consumption are shown in Supplementary Figure S1.

### 3.2. Orange Supplementation Does Not Affect Body Composition or Key Cardiometabolic Markers 

After 4 weeks of orange supplementation, body weight, waist circumference, and body composition measured by BIA (fat mass and fat-free mass) did not change (Table 3).

**Figure 2:**
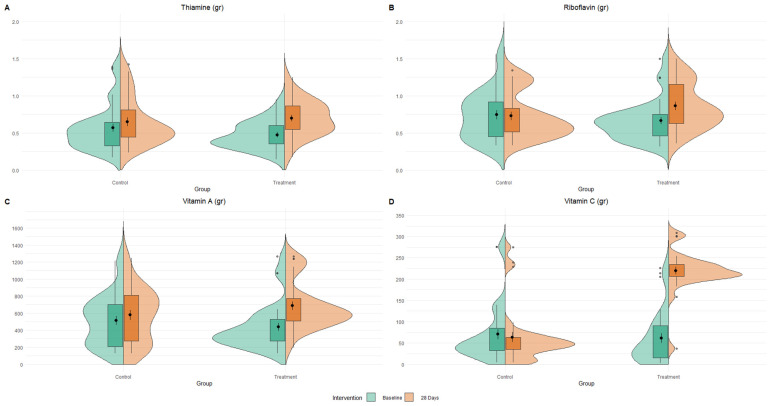
Changes in specific nutrient intake. Split-violin plots of vitamins, thiamine (**A**), riboflavin (**B**), vitamin A (**C**), and vitamin C (**D**) distribution between treatment time and groups.

Analysis of multiple 7-day food diaries indicated no significant change in daily caloric intake before and after the 28-day period (2026.91 ± 230.67 vs. 2036.65 ± 242.16, respectively, *p* = 0.15). Analysis of 7-day food diaries showed a significant increase in several key vitamins, including vitamin C, vitamin A, thiamine, and riboflavin, in the treatment group (Figure 2). 

Plasma substrates and hormones (glucose, insulin, and plasma lipids), markers of inflammation (C-reactive protein [CRP] and ferritin), and the homeostasis model assessment of insulin resistance (HOMA-IR) score did not change after orange supplementation (Table 4).

### 3.3. Orange Supplementation Reduces Liver Steatosis 

**Figure 3:**
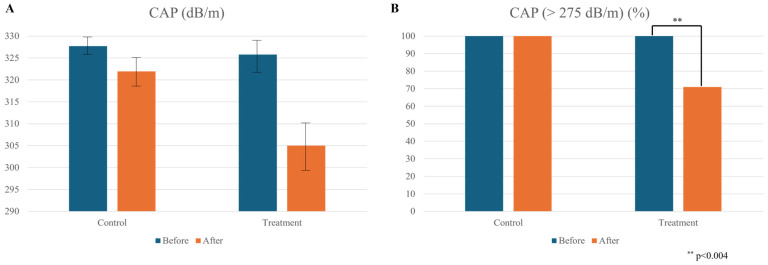
Impact of orange supplementation on liver steatosis. (**A**) Reduction in liver steatosis after four weeks of orange supplementation, as measured by controlled attenuation parameter (CAP = dB/m); (**B**) prevalence of liver steatosis based on CAP categorical analysis using a clinical cutoff of more than 275 dB/m [7]. Values are mean ± SEM.

After four weeks of orange supplementation, we observed a reduction in liver steatosis as measured by CAP as a categorical variable (Figure 3B, 70.97% vs. 100.00%, *p* < 0.004), although this did not reach statistical significance in a continuous manner (Figure 3A).

When analyzing CAP as a categorical variable based on the clinical cutoff of 275 dB/m [7], we found a significant decrease in the prevalence of subjects in the treatment group (Figure 3, panel B), with 70.97% of participants showing liver steatosis compared to 100% in the control group (*p* < 0.004). This indicates a reduction in liver disease prevalence of approximately 30% attributable to orange supplementation. However, no statistically significant changes in fibrosis degree (kPa) were observed in either group after the four-week period. Plasma concentrations of AST, ALT, and alkaline phosphates remained unchanged after orange supplementation (Table 3). However, there was a significant reduction in plasma GGT levels (Table 3).

## 4. Discussion

In this randomized clinical trial, unlike others focusing on orange juice consumption [11], we investigated the effects of supplementation with whole oranges, including the albedo rich in polyphenols [18], on liver function and steatosis using transient elastography. Over a four-week period, we evaluated these outcomes alongside various liver and cardiometabolic markers in weight-stable overweight individuals with MASLD and a CAP score exceeding 275 dB/m. Our findings indicate that daily consumption of 400 g of whole oranges—approximately four oranges per day—for 28 days significantly reduced the prevalence of liver steatosis, independent of any changes in body weight.

Lifestyle-induced weight loss leads to a dose-dependent improvement in liver function and steatosis, with reductions exceeding 10% of body weight achieving a 90% resolution rate of steatohepatitis [19]. Our findings highlight the potential of specific foods rich in phytochemicals and antioxidant vitamins, such as whole fruits like oranges, to enhance liver function as an adjunct treatment for MASLD. The significant reduction in liver steatosis prevalence, independent of changes in body weight and adiposity, is consistent with preclinical research suggesting that the flavonoids, vitamin C, and riboflavin found in oranges may support liver function [12,13]. However, unlike preclinical studies, our data indicate that these compounds can promote liver health independently of weight loss or improvements in insulin sensitivity, inflammation, and glucose and lipid metabolism, which typically require a negative energy balance [20,21,22].

This effect may be achieved by activating lipolytic and lipid oxidation pathways, such as PPAR-α [23,24], while simultaneously inhibiting de novo lipogenesis through mechanisms involving Liver X receptor (LXR-α) [25,26] and the dimethylarginine dimethylaminohydrolase (DDAH)/asymmetric dimethylarginine (ADMA) pathway [13].

Numerous studies have shown that oranges are rich in flavonoids and anthocyanins, which can positively influence lipid metabolism [27,28,29]. Key components such as polymethoxyflavones, narirutin, naringenin, tangerine, and hesperidin have lipid-lowering and antioxidant properties, preventing liver lipid accumulation and subsequent portal inflammation [29,30]. Other polyphenol-rich fruits like pomegranate and lychee have also been shown to reduce hepatic steatosis and insulin resistance in rodents, likely due to alterations in gut microbiota [31,32,33]. Additionally, increased intake of antioxidant vitamin C and thiamine may further enhance liver health by reducing liver oxidative stress [34,35,36]. Riboflavin is also involved in mitochondrial energy production, reducing the risk of fat accumulation in the liver [37].

Therapeutical benefits of the fruit-derived components have been demonstrated in the onset and progression of NAFLD [38,39]. The preventive effect of orange and pomelo peel powder on NAFLD has been demonstrated in mice through the reduction in HFD-induced dyslipidemia with a positive effect on liver inflammation [40]. Pomegranate fruit pulp has recently been shown to reduce hepatic steatosis and insulin resistance in mice by the modulation of the gut microbiota [41].

In subjects with metabolic syndrome, a high content of polyunsaturated fatty acids, tocopherols, and phenolic compounds in the diet led to reduced insulin resistance and glucose levels, improving lipid parameters and modulating the leptin and adiponectin levels in the serum [42].

Consequently, dietary components can largely determine the success of nutritional interventions in patients with metabolic diseases [39]. For example, the Mediterranean diet is a gold benchmark to treat MAFLD for its ability to reduce body weight, BMI, hip circumference, fat mass, and hypertension [43].

In a recent study conducted on tissue-engineered fatty liver, naringenin, found in many citrus fruits such as oranges, demonstrated a potential NAFLD-ameliorative property by decreasing fatty acid absorption and de novo lipogenesis and enhancing fatty acid oxidation [44]. Recently, naringenin has been reported to inhibit the NOD-like receptor protein 3 (NLRP3)/ nuclear factor-kappaB (NF-κB) pathway in a methionine-choline deficient (MCD) model of mice as well as in hepatoma carcinoma (HepG2) cells, primary hepatocytes, and Kupffer cells (KCs) [45].

The study has some limitations, including a small sample size and the absence of liver biopsies for histopathological and mechanistic characterization. The major strengths of this study include the intention-to-treat randomized controlled trial design minimizing the potential for selection bias and the high retention rate of enrolled participants with excellent adherence to the study intervention. A notable distinction of this study compared to previous research is the selection criteria for participants. Unlike earlier studies, our subjects were specifically chosen as overweight individuals with CAP values greater than 256 dB/m. This criterion ensures that all participants had a quantifiable degree of liver steatosis, making the study’s results more applicable to a clearly defined population of individuals with MASLD. This focused selection enhances the relevance and applicability of our findings to clinical practice for managing MASLD in overweight individuals.

## 5. Conclusions

Our findings underscore the potential effectiveness of whole fruit consumption, particularly citrus fruits, as a dietary strategy for reducing liver steatosis in overweight individuals with MASLD, independent of weight loss. This research adds to the growing evidence that fruits rich in vitamins, phytochemicals, and fiber can serve as adjunct therapy for preventing liver steatosis and MASLD. Future studies should explore the long-term effects of orange supplementation on fibrosis progression and overall metabolic health, as well as identify the specific bioactive compounds and microbial metabolites responsible for these benefits. Our study also suggests that daily consumption of whole oranges, including the albedo, as part of a balanced, moderately energy-restricted diet, combined with regular exercise, can be an effective preventive measure to reduce liver steatosis.

## Figures and Tables

**Table 1:** Physicochemical properties, polyphenol content, and total antioxidant activity of “Naveline” oranges.

Physicochemical Properties of Navelina Orange	
Fruit weight (g)	192.85 ± 25.59
Equatorial diameter (mm)	74.83 ± 2.14
Fruit height (mm)	75.83 ± 3.43
pH	3.46 ± 0.17
TA (g citric acid/L)	8.92 ± 1.14
TSS (° Brix)	9.75 ± 0.57
Maturity index (TSS/TAA)	11.09 ± 1.70
Polyphenols and total antioxidant activity of Navelina orange	
Total phenolic content (mg/kg flesh tissue)	1061.1 ± 136.8
Total phenolic content (mg/L juice)	134.0 ± 12.00
Hesperidin mg/L juice	505.7 ± 51.60
DPPH (mM TE/L juice)	7.90 ± 0.20
DPPH (mM TE/kg FW)	63.20 ± 3.70
ORAC (mM TE/kg FW)	155.40 ± 12.10

As mean and standard deviation (M ± SD).

**Table 2:** Baseline study subjects’ characteristics (*n* = 62).

Parameters ^1^	Control (*n* = 31)	Treatment (*n* = 31)	*p* ^2^
Age (yrs)	50.06 ± 9.77	51.77 ± 10.31	0.45
Gender (M) (%)	21 (67.74)	24 (77.42)	0.39 ^3^
*Anthropometric parameters*			
Weight (kg)	91.95 ± 11.42	91.98 ± 9.96	0.99
BMI (kg/m^2^)	32.31 ± 4.14	32.07 ± 4.25	0.97
Neck circumference (cm)	42.36 ± 11.17	39.61 ± 8.05	0.91
Waist circumference (cm)	108.60 ± 12.83	108.32 ± 12.72	0.53
Hip circumference (cm)	112.88 ± 10.02	109.77 ± 23.88	0.76
Whole body phA°	6.43 ± 0.66	6.11 ± 1.76	0.99
FFM (kg)	62.45 ± 9.50	58.40 ± 17.89	0.81
FM (kg)	30.49 ± 10.34	29.59 ± 15.94	0.44
*Biochemical parameters*			
Total cholesterol (mg/dL)	183.26 ± 42.88	202.29 ± 40.25	0.05
HDL cholesterol (mg%)	51.44 ± 10.43	47.10 ± 13.15	0.05
LDL cholesterol (mg/dL)	119.18 ± 39.86	132.11 ± 37.64	0.16
Triglycerides (mg/dL)	121.45 ± 70.41	132.10 ± 53.32	0.14
Fasting glucose (mg/GI)	94.71 ± 9.45	101.00 ± 21.62	0.27
Fasting insulin (µUI/mL)	15.24 ± 7.03	16.08 ± 8.55	0.58
HOMA-IR	3.58 ± 2.13	4.10 ± 2.50	0.39
AST (U/L)	23.48 ± 7.83	23.42 ± 9.93	0.70
ALT (U/L)	30.35 ± 15.74	36.68 ± 23.74	0.20
GGT (U/L)	29.71 ± 10.78	40.48 ± 23.01	0.05
Alkaline phosphatase (U/L)	69.77 ± 21.00	67.64 ± 19.99	0.76
CRP (mg/dL)	0.29 ± 0.23	0.34 ± 0.43	0.52
Ferritin (ng/mL)	191.35 ± 95.77	241.05 ± 170.98	0.48

^1^ As mean and standard deviation for continuous variables and as frequency and percentage (%) for categorical. ^2^ Wilcoxon rank-sum test (Mann–Whitney) or ^3^ Chi-square test where necessary. Abbreviations: BMI, body mass index; Rz, resistance; XC, reactance; phA, phase angle; BCM, body cell mass; FFM, free fat mass; FM, fat mass; TBW, total body water; ECW, extracellular water; CAP, controlled attenuation parameter; RBC, red blood cell; AST, Aspartate Aminotransferase; Alanine Transaminase; ALT, Alanine Transaminase; GGT, Gamma-Glutamyl Transferase; HDL, high-density lipoprotein; LDL, low-density lipoprotein; HOMA, homeostatic model assessment; CRP, C-reactive protein.

**Table 3:** Anthropometric parameters before and after intervention.

Parameters ^1^	Group		Between-Group ^2^
	Control	Treatment	
Weight (kg)			
Baseline	91.95 ± 11.42	91.98 ± 9.96	
28 Days	91.03 ± 11.47	91.26 ± 9.53	
Change	−0.010 ± 0.017	−0.007 ± 0.021	0.43
Within-Group ^3^	0.30	0.26	
BMI (kg/m^2^)			
Baseline	32.31 ± 4.14	32.07 ± 4.25	
28 Days	31.93 ± 4.35	31.95 ± 4.28	
Change	−0.012 ± 0.019	−0.003 ± 0.030	0.17
Within-Group ^3^	0.42	0.57	
Neck Circumference (cm)			
Baseline	42.36 ± 11.17	39.61 ± 8.05	
28 Days	39.78 ± 3.54	39.27 ± 8.04	
Change	−0.035 ± 0.110	−0.044 ± 0.182	0.19
Within-Group ^3^	0.07	0.15	
Waist circumference (cm)			
Baseline	108.60 ± 12.83	108.32 ± 12.72	
28 Days	107.57 ± 12.51	107.32 ± 12.13	
Change	−0.009 ± 0.015	−0.009 ± 0.018	0.91
Within-Group ^3^	0.45	0.05	
Hip circumference (cm)			
Baseline	112.88 ± 10.02	109.77 ± 23.88	
28 Days	112.19 ± 9.85	111.86 ± 11.30	
Change	−0.006 ± 0.014	−0.010 ± 0.023	0.68
Within-Group ^3^	0.04	0.04	
Whole Body phA°			
Baseline	6.43 ± 0.66	6.11 ± 1.76	
28 Days	6.48 ± 0.75	6.32 ± 1.39	
Change	0.009 ± 0.060	0.004 ± 0.069	0.56
Within-Group ^3^	0.56	0.85	
FFM (kg)			
Baseline	62.45 ± 9.50	58.40 ± 17.89	
28 Days	62.38 ± 10.08	61.19 ± 15.53	
Change	−0.002 ± 0.042	0.013 ± 0.053	0.49
Within-Group ^3^	0.14	0.58	
FM (kg)			
Baseline	30.49 ± 10.34	29.59 ± 15.94	
28 Days	29.37 ± 10.80	28.99 ± 13.36	
Change	−0.041 ± 0.116	−0.032 ± 0.097	0.97
Within-Group ^3^	0.14	0.14	

^1^ As mean and standard deviation for continuous variables and as frequency and percentage (%) for categorical. ^2^ Wilcoxon rank-sum test (Mann–Whitney); ^3^ Wilcoxon matched-pairs signed-rank test. Abbreviations: BMI, body mass index; Rz, resistance; XC, reactance; phA, phase angle; BCM, body cell mass; FFM, free fat mass; FM, fat mass.

**Table 4:** Metabolic parameters before and after intervention.

Parameters ^1^	Group		Between-Group ^2^
	Control	Treatment	
Total Cholesterol (mg/dL)			
Baseline	183.26 ± 42.88	202.29 ± 40.25	
28 Days	186.03 ± 35.94	193.39 ± 40.83	
Change	0.033 ± 0.134	−0.033 ± 0.145	0.07
Within-Group ^3^	0.85	0.28	
LDL Cholesterol (mg/dL)			
Baseline	119.18 ± 39.86	132.11 ± 37.64	
28 Days	121.22 ± 39.23	130.94 ± 35.58	
Change	0.046 ± 0.217	0.012 ± 0.160	0.69
Within-Group ^3^	0.10	0.47	
HDL Cholesterol (mg/dL)			
Baseline	51.44 ± 10.43	47.10 ± 13.15	
28 Days	51.80 ± 11.94	47.53 ± 10.74	
Change	0.007 ± 0.101	0.034 ± 0.173	0.92
Within-Group ^3^	0.20	0.72	
Triglycerides (mg/dL)			
Baseline	121.45 ± 70.41	132.10 ± 53.32	
28 Days	112.29 ± 70.34	123.06 ± 53.55	
Change	−0.056 ± 0.257	−0.032 ± 0.267	0.66
Within-Group ^3^	0.14	0.72	
Fasting Glucose (mg/dL)			
Baseline	94.71 ± 9.45	101.00 ± 21.62	
28 Days	95.71 ± 9.22	99.59 ± 26.83	
Change	0.012 ± 0.057	−0.018 ± 0.074	0.09
Within-Group ^3^	0.18	0.72	
Fasting Insulin (µUI/mL)			
Baseline	15.24 ± 7.03	16.08 ± 8.55	
28 Days	15.53 ± 8.21	16.42 ± 9.06	
Change	0.036 ± 0.317	0.067 ± 0.294	0.58
Within-Group ^3^	0.71	0.36	
HOMA-IR			
Baseline	3.58 ± 2.13	4.10 ± 2.50	
28 Days	3.63 ± 2.22	4.19 ± 2.94	
Change	0.055 ± 0.322	0.058 ± 0.337	0.94
Within-Group ^3^	0.99	0.99	
AST (U/L)			
Baseline	23.48 ± 7.83	23.42 ± 9.93	
28 Days	22.90 ± 7.45	24.29 ± 7.29	
Change	−0.010 ± 0.141	0.088 ± 0.241	0.11
Within-Group ^3^	0.99	0.05	
ALT (U/L)			
Baseline	30.35 ± 15.74	36.68 ± 23.74	
28 Days	29.93 ± 15.16	34.93 ± 18.50	
Change	−0.001 ± 0.138	0.008 ± 0.317	0.45
Within-Group ^3^	0.42	0.28	
GGT (U/L)			
Baseline	29.71 ± 10.78	40.48 ± 23.01	
28 Days	28.29 ± 10.31	34.22 ± 21.68	
Change	−0.041 ± 0.113	−0.160 ± 0.206	0.005
Within-Group ^3^	0.12	0.0001	
Alkaline Phosphatase (U/L)			
Baseline	69.77 ± 21.00	67.64 ± 19.99	
28 Days	71.58 ± 21.42	68.74 ± 19.88	
Change	0.029 ± 0.067	0.019 ± 0.069	0.66
Within-Group ^3^	0.56	0.58	
hsCRP (mg/dL)			
Baseline	0.29 ± 0.23	0.34 ± 0.43	
28 Days	0.30 ± 0.31	0.30 ± 0.41	
Change	0.201 ± 1.365	0.090 ± 0.740	0.79
Within-Group ^3^	0.03	0.58	
Ferritin (ng/mL)			
Baseline	191.35 ± 95.77	241.05 ± 170.98	
28 Days	188.07 ± 92.61	231.52 ± 163.94	
Change	−0.001 ± 0.155	−0.046 ± 0.206	0.32
Within-Group ^3^	0.85	0.15	

^1^ As mean and standard deviation for continuous variables and as frequency and percentage (%) for categorical. ^2^ Wilcoxon rank-sum test (Mann–Whitney); ^3^ Wilcoxon matched-pairs signed-rank test. Abbreviations: LDL, low-density lipoprotein; HDL, high-density lipoprotein; HOMA-IR, homeostatic model assessment for insulin resistance; AST, Aspartate Aminotransferase; ALT, Alanine Transaminase; GGT, Gamma-Glutamyl Transferase; hsCRP, high-sensitivity C-reactive protein.

## Data Availability

The original contributions presented in the study are included in the article. Further inquiries can be directed to the corresponding author.

## Supplementary Materials

The following supporting information can be downloaded at: https://www.mdpi.com/article/10.3390/nu16183191/s1, Table S1: List of foods rich in vitamin C.; Figure S1: Macronutrients and daily calorie consumption.

## References

[1] Lippolis T., Cofano M., Caponio G.R., De Nunzio V., Notarnicola M. (2023). Bioaccessibility and Bioavailability of Diet Polyphenols and Their Modulation of Gut Microbiota. Int. J. Mol. Sci..

[2] Assini J.M., Mulvihill E.E., Huff M.W. (2013). Citrus Flavonoids and Lipid Metabolism. Curr. Opin. Lipidol..

[3] Caponio G.R., Cofano M., Lippolis T., Gigante I., De Nunzio V., Difonzo G., Noviello M., Tarricone L., Gambacorta G., Giannelli G. (2022). Anti-Proliferative and Pro-Apoptotic Effects of Digested Aglianico Grape Pomace Extract in Human Colorectal Cancer Cells. Molecules.

[4] Fiore A., La Fauci L., Cervellati R., Guerra M.C., Speroni E., Costa S., Galvano G., De Lorenzo A., Bacchelli V., Fogliano V. (2005). Antioxidant Activity of Pasteurized and Sterilized Commercial Red Orange Juices. Mol. Nutr. Food Res..

[5] Grande F., Occhiuzzi M.A., Perri M.R., Ioele G., Rizzuti B., Statti G., Garofalo A. (2021). Polyphenols from Citrus Tacle® Extract Endowed with HMGCR Inhibitory Activity: An Antihypercholesterolemia Natural Remedy. Molecules.

[6] Weisberg S.P., McCann D., Desai M., Rosenbaum M., Leibel R.L., Ferrante A.W. (2003). Obesity Is Associated with Macrophage Accumulation in Adipose Tissue. J. Clin. Investig..

[7] Xu H., Barnes G.T., Yang Q., Tan G., Yang D., Chou C.J., Sole J., Nichols A., Ross J.S., Tartaglia L.A. (2003). Chronic Inflammation in Fat Plays a Crucial Role in the Development of Obesity-Related Insulin Resistance. J. Clin. Investig..

[8] Subramanian S., Goodspeed L., Wang S., Kim J., Zeng L., Ioannou G.N., Haigh W.G., Yeh M.M., Kowdley K.V., O’Brien K.D. (2011). Dietary Cholesterol Exacerbates Hepatic Steatosis and Inflammation in Obese LDL Receptor-Deficient Mice. J. Lipid Res..

[9] Subramanian S., Han C.Y., Chiba T., McMillen T.S., Wang S.A., Haw A., Kirk E.A., O’Brien K.D., Chait A. (2008). Dietary Cholesterol Worsens Adipose Tissue Macrophage Accumulation and Atherosclerosis in Obese LDL Receptor–Deficient Mice. Arter. Thromb. Vasc. Biol..

[10] McNeill K., Fontana L., Ritter J., Russel-Jones D., Chowienczyk P. (1998). Inhibitory Effects of Low-Density Lipoprotein on Endothelium-Dependent Relaxation Are Exaggerated in Men with NIDDM. Diabetic. Med..

[11] Dos Santos K.G., Yoshinaga M.Y., Glezer I., de Britto Chaves-Filho A., de Santana A.A., Kovacs C., Magnoni C.D., Lajolo F.M., Miyamoto S., Aymoto Hassimotto N.M. (2022). Orange Juice Intake by Obese and Insulin-Resistant Subjects Lowers Specific Plasma Triglycerides: A Randomized Clinical Trial. Clin. Nutr. ESPEN.

[12] Salamone F. (2012). Moro Orange Juice Prevents Fatty Liver in Mice. World J. Gastroenterol..

[13] Sorrenti V., Di Giacomo C., Acquaviva R., Cosenza J., Carota G., Galvano F. (2019). Blond and Blood Juice Supplementation in High Fat Diet Fed Mice: Effect on Antioxidant Status and DDAH/ADMA Pathway. RSC Adv..

[14] Kaya E., Yilmaz Y. (2022). Epidemiology, Natural History, and Diagnosis of Metabolic Dysfunction-Associated Fatty Liver Disease: A Comparative Review with Nonalcoholic Fatty Liver Disease. Ther. Adv. Endocrinol. Metab..

[15] Day C.P., James O.F.W. (1998). Steatohepatitis: A Tale of Two “Hits”?. Gastroenterology.

[16] Eslam M., Newsome P.N., Sarin S.K., Anstee Q.M., Targher G., Romero-Gomez M., Zelber-Sagi S., Wai-Sun Wong V., Dufour J.-F., Schattenberg J.M. (2020). A New Definition for Metabolic Dysfunction-Associated Fatty Liver Disease: An International Expert Consensus Statement. J. Hepatol..

[17] Boccatonda A., Andreetto L., D’Ardes D., Cocco G., Rossi I., Vicari S., Schiavone C., Cipollone F., Guagnano M.T. (2023). From NAFLD to MAFLD: Definition, Pathophysiological Basis and Cardiovascular Implications. Biomedicines.

[18] Smeriglio A., Cornara L., Denaro M., Barreca D., Burlando B., Xiao J., Trombetta D. (2019). Antioxidant and Cytoprotective Activities of an Ancient Mediterranean Citrus (*Citrus Lumia Risso*) Albedo Extract: Microscopic Observations and Polyphenol Characterization. Food Chem..

[19] Vilar-Gomez E., Martinez-Perez Y., Calzadilla-Bertot L., Torres-Gonzalez A., Gra-Oramas B., Gonzalez-Fabian L., Friedman S.L., Diago M., Romero-Gomez M. (2015). Weight Loss Through Lifestyle Modification Significantly Reduces Features of Nonalcoholic Steatohepatitis. Gastroenterology.

[20] Kraus W., Bhapkar M., Huffman K.M., Pieper C.F., Das S.K., Redman L.M., Villareal D.T., Rochon J., Roberts S.B., Ravussin E. (2019). 2 Years of Calorie Restriction and Cardiometabolic Risk (CALERIE): Exploratory Outcomes of a Multicentre, Phase 2, Randomised Controlled Trial. Lancet Diabetes Endocrinol..

[21] Green C.L., Lamming D.W., Fontana L. (2022). Molecular Mechanisms of Dietary Restriction Promoting Health and Longevity. Nat. Rev. Cell Biol..

[22] Ruggenenti P., Abbate M., Ruggiero B., Rota S., Trillini M., Aparicio C., Parvanova A., Petrov Iliev I., Pisanu G., Perna A. (2017). Renal and Systemic Effects of Calorie Restriction in Patients with Type 2 Diabetes with Abdominal Obesity: A Randomized Controlled Trial. Diabetes.

[23] Hashimoto T., Cook W.S., Qi C., Yeldandi A.V., Reddy J.K., Rao M.S. (2000). Defect in Peroxisome Proliferator-Activated Receptor α-Inducible Fatty Acid Oxidation Determines the Severity of Hepatic Steatosis in Response to Fasting. J. Biol. Chem..

[24] Seo Y.S., Kim J.H., Jo N.Y., Choi K.M., Baik S.H., Park J., Kim J.S., Byun K.S., Bak Y., Lee C.H. (2008). PPAR Agonists Treatment Is Effective in a Nonalcoholic Fatty Liver Disease Animal Model by Modulating Fatty-acid Metabolic Enzymes. J. Gastroenterol. Hepatol..

[25] Kay H.Y., Kim W.D., Hwang S.J., Choi H.-S., Gilroy R.K., Wan Y.-J.Y., Kim S.G. (2011). Nrf2 Inhibits LXRα-Dependent Hepatic Lipogenesis by Competing with FXR for Acetylase Binding. Antioxid. Redox Signal.

[26] Kim Y.W., Kim Y.M., Yang Y.M., Kim T.H., Hwang S.J., Lee J.R., Kim S.C., Kim S.G. (2010). Inhibition of SREBP-1c-Mediated Hepatic Steatosis and Oxidative Stress by Sauchinone, an AMPK-Activating Lignan in Saururus Chinensis. Free Radic. Biol. Med..

[27] Titta L., Trinei M., Stendardo M., Berniakovich I., Petroni K., Tonelli C., Riso P., Porrini M., Minucci S., Pelicci P.G. (2010). Blood Orange Juice Inhibits Fat Accumulation in Mice. Int. J. Obes..

[28] FFujimori A.S., Ribeiro A.P., Pereira A.G., Dias-Audibert F.L., Tonon C.R., Dos Santos P.P., Dantas D., Zanati S.G., Catharino R.R., Zornoff L.A. (2023). Effects of Pera Orange Juice and Moro Orange Juice in Healthy Rats: A Metabolomic Approach. Metabolites.

[29] Pan M., Yang G., Li S., Li M., Tsai M., Wu J., Badmaev V., Ho C., Lai C. (2017). Combination of Citrus Polymethoxyflavones, Green Tea Polyphenols, and Lychee Extracts Suppresses Obesity and Hepatic Steatosis in High-fat Diet Induced Obese Mice. Mol. Nutr. Food Res..

[30] Tsuda T., Horio F., Uchida K., Aoki H., Osawa T. (2003). Dietary Cyanidin 3-O-β-D-Glucoside-Rich Purple Corn Color Prevents Obesity and Ameliorates Hyperglycemia in Mice. J. Nutr..

[31] Tangestani H., Jamshidi A., Farhadi A., Ghalandari H., Dehghani P., Moghaddas N., Safaei Z., Emamat H. (2024). The Effects of Pomegranate (*Punica granatum*) on Nonalcoholic Fatty Liver Disease: A Systematic Review of in Vivo Interventional Studies. Phytother. Res..

[32] Sánchez-Terrón G., Martínez R., Morcuende D., Caballero V., Estévez M. (2024). Pomegranate Supplementation Alleviates Dyslipidemia and the Onset of Non-Alcoholic Fatty Liver Disease in Wistar Rats by Shifting Microbiota and Producing Urolithin-like Microbial Metabolites. Food Funct..

[33] Jinato T., Chayanupatkul M., Dissayabutra T., Chutaputti A., Tangkijvanich P., Chuaypen N. (2022). Litchi-Derived Polyphenol Alleviates Liver Steatosis and Gut Dysbiosis in Patients with Non-Alcoholic Fatty Liver Disease: A Randomized Double-Blinded, Placebo-Controlled Study. Nutrients.

[34] He Z., Li X., Yang H., Wu P., Wang S., Cao D., Guo X., Xu Z., Gao J., Zhang W. (2021). Effects of Oral Vitamin C Supplementation on Liver Health and Associated Parameters in Patients with Non-Alcoholic Fatty Liver Disease: A Randomized Clinical Trial. Front. Nutr..

[35] Lee H., Ahn J., Shin S.S., Yoon M. (2019). Ascorbic Acid Inhibits Visceral Obesity and Nonalcoholic Fatty Liver Disease by Activating Peroxisome Proliferator-Activated Receptor α in High-Fat-Diet-Fed C57BL/6J Mice. Int. J. Obes..

[36] Kalyesubula M., Mopuri R., Asiku J., Rosov A., Yosefi S., Edery N., Bocobza S., Moallem U., Dvir H. (2021). High-Dose Vitamin B1 Therapy Prevents the Development of Experimental Fatty Liver Driven by Overnutrition. Dis. Model. Mech..

[37] Wang Y., Bian X., Wan M., Dong W., Gao W., Yao Z., Guo C. (2024). Effects of Riboflavin Deficiency and High Dietary Fat on Hepatic Lipid Accumulation: A Synergetic Action in the Development of Non-Alcoholic Fatty Liver Disease. Nutr. Metab..

[38] Pathak M.P., Pathak K., Saikia R., Gogoi U., Patowary P., Chattopadhyay P., Das A. (2023). Therapeutic Potential of Bioactive Phytoconstituents Found in Fruits in the Treatment of Non-Alcoholic Fatty Liver Disease: A Comprehensive Review. Heliyon.

[39] Beygi M., Ahi S., Zolghadri S., Stanek A. (2024). Management of Metabolic-Associated Fatty Liver Disease/Metabolic Dysfunction-Associated Steatotic Liver Disease: From Medication Therapy to Nutritional Interventions. Nutrients.

[40] Hu M., Zhang L., Ruan Z., Han P., Yu Y. (2021). The Regulatory Effects of Citrus Peel Powder on Liver Metabolites and Gut Flora in Mice with Non-Alcoholic Fatty Liver Disease (NAFLD). Foods.

[41] Song H., Shen X., Chu Q., Zheng X. (2022). Pomegranate Fruit Pulp Polyphenols Reduce Diet-induced Obesity with Modulation of Gut Microbiota in Mice. J. Sci. Food Agric..

[42] Silva Figueiredo P., Carla Inada A., Marcelino G., Maiara Lopes Cardozo C., De Cássia Freitas K., De Cássia Avellaneda Guimarães R., Pereira de Castro A., Aragão do Nascimento V., Aiko Hiane P. (2017). Fatty Acids Consumption: The Role Metabolic Aspects Involved in Obesity and Its Associated Disorders. Nutrients.

[43] Tsitsou S., Cholongitas E., Bali T., Neonaki A., Poulia K.-A., Papakonstantinou E. (2024). Effects of Time-Restricted Hypocaloric Mediterranean Diet in Patients with Non-Alcoholic Fatty Liver Disease: Preliminary Data from the CHRONO-NAFLD Project. Proceedings.

[44] Zhang X., Zhang Y., Gao W., Guo Z., Wang K., Liu S., Duan Z., Chen Y. (2021). Naringin Improves Lipid Metabolism in a Tissue-Engineered Liver Model of NAFLD and the Underlying Mechanisms. Life Sci..

[45] Wang Q., Ou Y., Hu G., Wen C., Yue S., Chen C., Xu L., Xie J., Dai H., Xiao H. (2020). Naringenin Attenuates Non-alcoholic Fatty Liver Disease by Down-regulating the NLRP3/NF-κB Pathway in Mice. Br. J. Pharmacol..

