# Supplementary material for: Daily Orange Consumption Reduces Hepatic Steatosis Prevalence in Patients with Metabolic Dysfunction-Associated Steatotic Liver Disease: Exploratory Outcomes of a Randomized Clinical Trial
Source: Nutrients. 2024 Sep 20;16(18):3191. doi: 10.3390/nu16183191 (PMC11435367; doi:10.3390/nu16183191)
Supplement: Supplementary file 1 [file nutrients-16-03191-s001.zip › Supplementary Material(1).pdf]

**Supplementary Table S1.** List of foods rich in vitamin C.

| Grains | Nuts | Fruits | Vegetables | Herbs/Spices/Condiments | Drinks | Others |
|---|---|---|---|---|---|---|
| Gentle Red Wheat | Almonds | Apple | Artichokes | Basil | Chinotto | Citrus Jams and Preserves |
| Sorghum | Nuts | Apricots | Broccoli | Chili | Citrus Flavored Tea | Preserves |
| | | Banana | Brussels sprouts | Mint | Fruit Juices | |
| | | Bergamot | Cabbage | Origan | Fruit Juices (All Types) | |
| | | Blackcurrant | Cantaloupe | Parsley | Lemonades | |
| | | Calamondino or Bitter Mandarin | Cauliflower | Peppermint | Red Wine | |
| | | Cedar | Endive | Rosemary | White Wine | |
| | | Chinese Mandarin or Kumquat | Potatoes | Valerian | | |
| | | Clementines | Spinach | | | |
| | | Combava or Kaffir Lime | | | | |
| | | Finger Limes | | | | |
| | | Grapefruit | | | | |
| | | Kiwi | | | | |
| | | Lemon | | | | |
| | | Lettuce | | | | |
| | | Lime | | | | |
| | | Mandarin | | | | |
| | | Mango | | | | |
| | | Mapo | | | | |
| | | Orange | | | | |
| | | Papaya | | | | |
| | | Peas | | | | |
| | | Peppers | | | | |
| | | Plums | | | | |
| | | Pomelo | | | | |
| | | Pumpkin | | | | |
| | | Raw Red Tomatoes | | | | |
| | | Strawberries | | | | |
| | | Tangelo | | | | |
| | | Tangerines | | | | |
| | | Tangor | | | | |

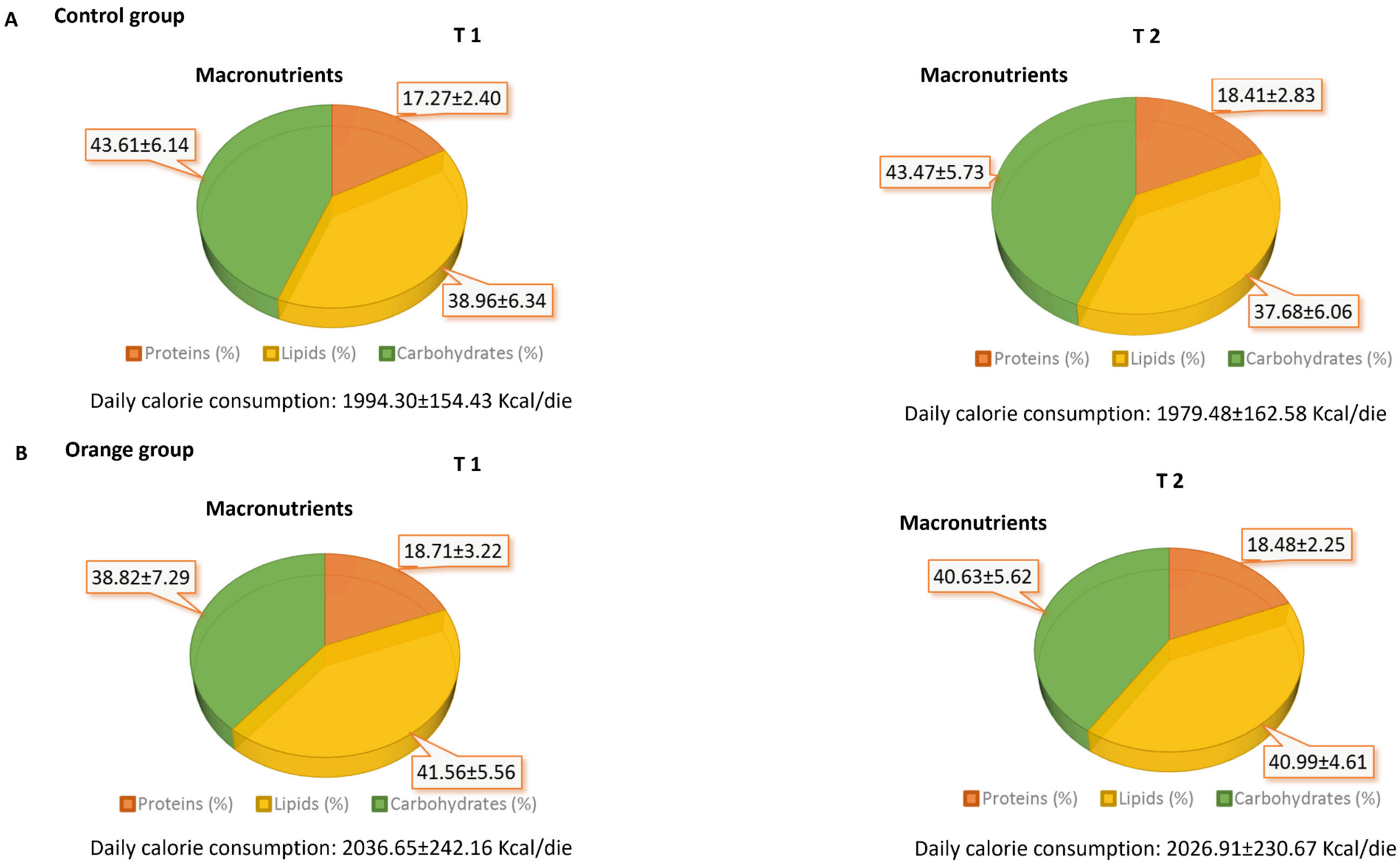


**Supplementary Figure S1.** Macronutrients and daily calories consumption in control and "orange" group.
